# *Ocimum sanctum* as a Source of Quorum Sensing Inhibitors to Combat Antibiotic Resistance of Human and Aquaculture Pathogens

**DOI:** 10.3390/life14070785

**Published:** 2024-06-21

**Authors:** Sybiya Vasantha Packiavathy Issac Abraham, Veera Ravi Arumugam, Nancy Immaculate Mary, Jeba Sweetly Dharmadhas, Rajamanikandan Sundararaj, Arul Ananth Devanesan, Ramachandran Rajamanickam, Raja Veerapandian, John Paul John Bosco, Jeyapragash Danaraj

**Affiliations:** 1 Division of Biotechnology, Karunya Institute of Technology and Sciences, Coimbatore 641 114, Tamil Nadu, India; 2 Department of Biotechnology, Alagappa University, Karaikudi 630 003, Tamil Nadu, India; aveeraravi@rediffmail.com (V.R.A.); nancybiotech@gmail.com (N.I.M.); 3 Department of Biochemistry and Biotechnology, Avinashilingam Institute for Home Science and Higher Education for Women, Coimbatore 641 043, Tamil Nadu, India; sweetly5647@gmail.com; 4 Centre for Drug Discovery, Karpagam Academy of Higher Education, Coimbatore 641 021, Tamil Nadu, India; mani.bioinfor@gmail.com; 5 Department of Biotechnology, The American College, Satellite Campus, Madurai 625 503, Tamil Nadu, India; d.arulananth@gmail.com; 6 Department of Biotechnology, Srimad Andavan Arts and Science College (Autonomous), Tiruchirappalli 620 005, Tamil Nadu, India; raramachandran.r@gmail.com; 7 Department of Molecular and Translational Medicine, Center of Emphasis in Infectious Diseases, Texas Tech University Health Sciences Center, El Paso, TX 79905, USA; rajja87@gmail.com; 8 Division of Electronics and Communication Engineering, Karunya Institute of Technology and Sciences, Coimbatore 641 114, Tamil Nadu, India; johnpaul@karunya.edu; 9 Centre for Ocean Research, Sathyabama Research Park, Sathyabama Institute of Science and Technology, Chennai 600 119, Tamil Nadu, India; pragashdb@gmail.com

**Keywords:** biofilms, antibiotic resistance, quorum sensing inhibition, *Ocimum sanctum*, eugenol

## Abstract

Biofilms play a decisive role in the infectious process and the development of antibiotic resistance. The establishment of bacterial biofilms is regulated by a signal-mediated cell–cell communication process called “quorum sensing” (QS). The identification of quorum sensing inhibitors (QSI) to mitigate the QS process may facilitate the development of novel treatment strategies for biofilm-based infections. In this study, the traditional medicinal plant *Ocimum sanctum* was screened for QS inhibitory potential. Sub-MICs of the extract significantly affected the secretion of EPS in Gram-negative human pathogens such as *Escherichia coli*, *Pseudomonas aeruginosa* PAO1, *Proteus mirabilis*, and *Serratia marcescens*, as well as aquaculture pathogens *Vibrio harveyi*, *V. parahaemolyticus*, and *V. vulnificus*, which render the bacteria more sensitive, leading to a loss of bacterial biomass from the substratum. The observed inhibitory activity of the *O. sanctum* extract might be attributed to the presence of eugenol, as evidenced through ultraviolet (UV)-visible, gas chromatography-mass spectroscopy (GC–MS), Fourier transformer infrared (FTIR) spectroscopy analyses, and computational studies. Additionally, the QSI potential of eugenol was corroborated through in vitro studies using the marker strain *Chromobacterium violaceum*.

## 1. Introduction

Naturally, microorganisms have the tendency to attach to biotic or abiotic surfaces, proliferate, and become immersed in a self-secreted slimy matrix composed of extracellular polymeric substances (EPS) to form a complex structure, termed a “biofilm” [1,2]. In the multistep process of biofilm development, the initial adherence of free-swimming bacteria to the substratum is the first step, followed by irreversible attachment and colonization. The EPS and water channels produced during this process help with the supply of nutrients, resulting in the maturation of biofilms [3]. Additionally, the sessile cells in EPS are more resistant to extreme environments than their planktonic counterparts. Thus, bacterial resistance becomes one of the characteristics of bacterial biofilm communities, which contributes to the virulence to the bacteria [4]. As reported by the National Institute of Health (NIH), microbial biofilms are responsible for approximately 80% of all human infections [5]. Indeed, the establishment of biofilms by *Pseudomonas aeruginosa*, *Escherichia coli*, *Serratia marcescens*, and *Proteus mirabilis* is considered as the major cause of a wide range of chronic, persistent, and catheter-associated infections in humans [2,4]. Moreover, several pathogenic *Vibrio* species are able to cause diseases in aquatic organisms and humans, which, in turn, exert a negative impact on environmental, economic, and public health [6]. The biofilm-forming potential of *Vibrio* spp. is considered as the root cause of their survival, virulence, and stress resistance [7]. 

Antibiotics are being employed in the treatment of biofilm-based infections in humans as well as aquatic organisms. However, the overuse of antibiotics will lead to the development of resistance, thus rendering antibiotic treatments ineffective [8]. The emergence of resistance imposes a major risk to society, bringing about difficulties in treating human and animal infections; this phenomenon is expected to worsen in the near future if proper control measures are not instigated.

Since biofilm formation, an important virulence factor, plays a key role in bacterial infections, preventing pathogens from producing them seems to be a suitable alternate therapeutic strategy for the control of bacterial diseases. One such alternate target in this aspect is the disruption of bacterial cell-to-cell communication, the so-called “quorum sensing” (QS) system in which bacteria coordinate the expression of certain genes in response to the presence of small molecules. QS molecules are found to regulate the expression of genes responsible for the formation and maturation of biofilms [9]. QS-mediated biofilm formation has been extensively studied in the bacterial pathogens involved in all implant-related infections in the medical field [10], in environmental biofouling [9], and in infections in aquatic organisms [8]. Unlike antibiotics, QS inhibitors will not put bacteria under strong selective pressure and are not likely to select for drug-resistant bacteria [11]. Therefore, attenuating QS-mediated biofilm formation and EPS production through QSI compounds could plausibly pave the way for the development of novel antibiofilm compounds. 

Plants employed in traditional medicinal treatments are considered as promising sources in the search for QSI compounds [12,13]. QSI activity at sub-lethal concentrations of dietary phytochemicals from plants known to have several health benefits and antimicrobial activity was documented by Vattem et al. [12]. Similarly, phytochemicals derived from dietary extracts, including fruit, herbs, and spices, were shown to have QS inhibitory effects against *Chromobacterium violaceum* 026 (CV026) and CV 31532 and *P. aeruginosa* (PAO1). Vanilla, a well-known spice, was found to mitigate the QS mechanism of *C. violaceum* [14]. Similarly, extracts of south Indian spices such as *Capparis spinosa* [15] and *Cuminum cyminum* [16] showed pronounced antibiofilm activity against human bacterial pathogens. *Ocimum sanctum*, known as Holy Basil, is a traditional medicinal plant with a wide range of bioactive compounds that contribute to its various pharmacological properties. Though this plant is well known for its medicinal properties, studies on its QSI activity against bacterial pathogens are largely lacking. Hence, the present study aims to investigate the QSI potential of *O. sanctum*.

## 2. Materials and Methods

### 2.1. Cultivation and Maintenance of Target Pathogens

The test bacterial strains used in this investigation, i.e., *Escherichia coli* (ATCC 10536), *Pseudomonas aeruginosa* PAO1, *Proteus mirabilis* (ATCC 7002), *Serratia marcescens* (FJ584421) and *Chromobacterium violaceum* (ATCC 12472) were cultivated and sub-cultured in Luria-Bertani (LB) medium. All the test human pathogens were maintained at 37 °C and the biomarker strain was grown at 30 °C. For aquatic pathogens, the strains *Vibrio harveyi (MTCC 3438)*, *V. parahaemolyticus* (ATCC 17802), and *V. vulnificus* (MTCC 1145) were cultured and maintained in marine Luria Bertani (mLB) broth (pH 7.5 ± 0.2) at 30 °C. For this study, all the test strains were routinely sub-cultured in LB medium until cell concentrations reached 1 × 10^8^ CFU/mL.

### 2.2. Preparation of Extracts

The extraction was carried out by following the method described by Packiavathy et al. [16]. Briefly, a cleaned leaf sample of *O. sanctum* was shade dried at room temperature and ground to a fine powder. To 5 g of powdered sample, 50 mL of methanol was added, and the mixture was incubated overnight at room temperature. The methanol phase was collected and the concentrated extract was transferred into small vials. It was then evaporated to dryness at 55 °C. Residues were weighed and dissolved in deionized water. The extracts were kept at 20 °C until they were used in bioassays.

### 2.3. Determination of Minimum Inhibitory Concentration (MIC)

The MIC of the *O. sanctum* extract was determined by following the guidelines suggested by the Clinical and Laboratory Standards Institute [17]. Briefly, 1% of test bacterial strains at 1 × 10^8^ CFU/mL density was added to an appropriate growth medium supplemented with twofold serially diluted test extract to achieve final concentrations ranging from 50 to 0.012 mg/mL. These samples were then allowed to grow at the respective temperature for 18–24 h. The MIC was considered as the minimum concentration that completely inhibited the visible growth of bacteria. 

### 2.4. Screening for QSI Activity

The methanolic extract of *O. sanctum* was screened for its QSI potential by inhibiting the QS mechanism of *C. violaceum* (ATCC 12472). It synthesizes a violet color pigment termed “violacein” in response to C6-HSL, the QS signal molecule produced by the AI synthase CviI. This signal binds to cognate receptor CviR to form a complex which, in turn, induces the genes responsible for violacein synthesis [18]. Briefly, 1% overnight culture (0.4 OD at 600 nm) of *C. violaceum* (ATCC 12472) was incubated with various concentrations of *O. sanctum* (3–12 mg/mL) in micro-titre plates (MTP) containing 1 mL of LB broth. Wells without extract served as controls and were incubated at 30 °C for 16 h.

### 2.5. Biofilm Biomass Quantification Assay

The biofilm inhibitory effect of the *O. sanctum* extract against target pathogens was assessed by measuring the biofilm biomass using microtiter plate (MTP) assay [16]. To 1% of test bacteria at 1 × 10^8^ CFU/mL concentration, 1 mL of growth medium was added without or with a varying concentration of *O. sanctum* extract. Bacteria were allowed to adhere and grow without agitation for 18 h at the appropriate temperature. After incubation, the spent media in MTPs was discarded, along with free-swimming planktonic cells, and the wells were gently washed twice with deionized water. The biofilm cells that adhered to the surface of MTP were stained with 200 µL of 0.4% crystal violet (CV) (Hi-media, Mumbai, India) solution. After staining with CV solution for 5 min, the content was discarded completely, and the wells were washed twice with deionized water to remove the excess stain. Subsequently, 1 mL of 95% ethanol was added to each well to elute CV from the stained cells. The biofilm biomass was directly proportional to the absorbance of the eluted solution at OD 650nm using a UV–visible spectrophotometer. The reduction in biofilm biomass was quantified by the formula as follows: Percentage inhibition of biofilm biomass = Control OD − Test OD/Control OD ×100

### 2.6. EPS Inhibition Assay

Determination of EPS was carried out via the total carbohydrate assay. Cover slips of 1 × 1 cm^2^ were immersed in the culture, untreated and treated with varying concentrations of *O. sanctum* extract in 24-wells of MTP, and incubated for 24 h. After incubation, the cover slips were carefully taken and washed with 0.5 mL of 0.9% NaCl. The cell suspensions in 0.5 mL of 0.9% NaCl were incubated with an equal volume of 5% phenol (0.5 mL) in test tubes, to which 5 volumes of concentrated H_2_SO_4_ were added. The mixture was then incubated for 1 h in the dark, followed by centrifugation at 10,000 rpm for 10 min. The absorbance of the resulting supernatant was read at 490 nm [16]. The reduction in EPS production was calculated as follows: Percentage inhibition of EPS = Control OD − Test OD/Control OD × 100.

### 2.7. Antibacterial Assay

The effect of the *O. sanctum* extracts on bacterial growth at the test concentrations was assessed through well diffusion agar assay (WDAA) using Muller-Hinton agar (MHA) (Hi-media, Mumbai, India), as described by the Clinical and Laboratory Standards Institute [17]. Briefly, 100 µL each of test bacteria *E. coli*, PAO1, *P. mirabilis*, *S. marcescens*, *Vibrio harveyi, V. parahaemolyticus*, and *V. vulnificus*, with 0.5 McFarland standard units (1 × 10^8^ CFU/mL), was uniformly spread over MHA plates. In order to absorb excess moisture, the plates were kept undisturbed for 10 min. Sterile paper disks (Hi-media, Mumbai, India) with a diameter of 10 mm, loaded with varying concentrations (3–12 mg/mL) of *O. sanctum* extracts, were added to the wells and incubated at 30 °C. After 24 h of incubation, the plates were monitored for a growth inhibition zone. 

### 2.8. Gas Chromatography—Mass Spectrum (GC-MS) Analysis

Firstly, 10 g of *O. sanctum* leaves were dried and powdered. They then underwent extraction with 100 mL of methanol in a rotatory shaker overnight at 37 °C. Subsequently, the crude extract was filtered using Whatman paper no.1, and the filtrate was dried at 50 °C under vacuum. The dried extract was scraped out, weighed to obtain percentage yields, and stored in sealed vials. The extract was resuspended with the required volume of methanol and analyzed with GC-MS to identify the active principle responsible for the QSI activity to *O. sanctum.* For GC-MS analysis, gas chromatography (GC-2010), interfaced with quadrupole mass spectrometer (QP-2010) (Shimadzu Corporation, Kyoto, Japan) analysis, was used to determine the chemical constituents of *O. sanctum* using Rtx-PCB capillary column (60 m × 0.25 mm i.d., 0.25 mm film thickness, RESTEK, Bellefonte, PA, USA). Helium with a purity of 99.99% was used as the carrier gas at a flow rate of 1 mL/min. One ml of extract was injected in split mode using an auto sampler. The injector port, interface, and ion source temperatures were set at 250, 270, and 230 °C, respectively. The GC temperature was programmed as follows: 50 °C (1 min), 10 °C/min ramp to 320 °C (10 min hold). The mass spectrometer was operated in electron ionization (EI) mode at 70 eV and at an emission current of 60 mA. Full scan data were obtained in a mass range of *m*/*z* 50–500. The identification of the compounds was based on 90% similarity between the MS spectra of unknown and reference compounds in a MS spectra library.

### 2.9. Column Chromatography 

For column chromatography, 100 mg of *O. sanctum* extract was loaded on to a 20-cm long glass column with a 2-cm diameter silica gel column (Silica gel 60–180 mesh, Sisco Research Laboratories, Mumbai, India). After adsorption, successive extractions of *O. sanctum* extract were carried out using 50 mL of hexane (fractions 1 and 2), 50 mL of petroleum ether (fractions 3 and 4), 100 mL of benzene (fractions 5 to 8), 100 mL benzene:chloroform (50:50) (fractions 9 to 12), 100 mL of chloroform (fractions 13 to 16), 100 mL of chloroform:ethyl acetate (50:50) (fractions 17 to 20), 100 mL of ethyl acetate (fractions 20 to 24), ethyl acetate:dichloromethane (50:50) (fractions 25 to 28), 100 mL of dichloromethane (fractions 29 to 32), dichloromethane:methanol (50:50) (fractions 33 to 36), 150 mL of methanol (fractions 37 to 42), and 100 mL of methanol:water (50:50) (fractions 43 to 46). Fractions of 25 mL each were evaporated to dryness before being resuspended with the required volume of methanol and examined for their QSI activity against violacein production using *C. violaceum* (ATCC 12472). The fractions which inhibited violacein production without affecting bacterial growth were considered to be the active fractions. The fractions showing QSI activity were further purified by using the same column chromatography protocol.

### 2.10. Computational Studies

To examine the structure–activity relationship between QS receptor protein RhlR of the universal marker strain *P.aeruginosa* and the major components of *O. sanctum*, such as eugenol and acetyl eugenol, the molecular dynamics were examined and molecular docking studies were performed [19]. The amino acid sequence of RhlR of *P. aeruginosa* was retrieved from the UniProt database using accession number P54292; this was used for homology modeling in the Modeller9v7 software. The model quality was further accessed via a Ramachandran plot. For docking studies, the protein was initially prepared using the Protein Preparation Wizard of Schrödinger 2021 (Schrödinger, LLC, New York, NY, USA) with the default setting. The optimization and minimization of the RhlR protein were carried out using the OPLS-2005 (Optimized Potential Liquid Simulation) force field. The chemical structures of acetyl eugenol and eugenol were downloaded from the PubChem database and prepared via the LigPrep module in Schrödinger 2021 (Schrödinger, LLC, New York, NY, USA) using the default setting. The active site information for the RhlR was obtained from the template structure, and the grid was generated around the active site of the amino acid using the Receptor Grid Generation Panel in Schrödinger. Finally, glide extra precision mode was used for molecular docking. 

The Desmond v3 program with an OPLS-2005 force field was used for molecular dynamics simulation of the RhlR-ligand complexes. The protein–ligand complex was simulated in a simple point charge water environment, and the system was brought to a neutral state by adding appropriate counter ions (Na^+^ and Cl^−^). The Particle Mesh Ewald technique was used to estimate long-range electrostatic interactions. Chain coupling techniques were used to achieve position-restrained equilibration under the NPT (constant number of particles, pressure, and temperature) ensemble at a constant temperature of 300 K and a pressure of 1 bar. Finally, a 100 nano-second molecular dynamic simulation was carried out, and the results were analyzed.

### 2.11. Fourier Transform—Infrared Spectra (FT-IR) Analysis

An FT-IR analysis was performed in order to identify the functional groups present in active fraction obtained from *O. sanctum*. The FT-IR spectra in the 400–4000 cm^−1^ region were recorded on a Tensor 27 FTIR Spectrometer equipped with DLaTGS detector (Bruker, Karlsruhe, Germany). The spectra were obtained using the potassium bromide (KBr) pellet technique. Potassium bromide (AR grade) was dried under vacuum at 100 °C for 48 h. Then, 100 mg of KBr with 1 mg of *O. sanctum* extract pellet was taken to prepare KBr pellets. The frequencies for all sharp bands were determined accurately from the original base line corrected spectra belonging to the corresponding groups. 

### 2.12. UV–Visible Spectrum Analysis

The GC–MS analysis in the present study, as well as other reported evidence [20,21] revealed the presence of eugenol as a major component in *O. sanctum*. To confirm this, the active fraction obtained through column chromatography, along with commercially available eugenol (Alfa Aesar, Ward Hill, MA, USA), were subjected to spectrum analyses in a UV range of 200–400 nm for comparison.

### 2.13. Confirmation of QSI Activity of Eugenol Using Universal Biomarker

The universal biomarker strain *Chromobacterium violaceum* (ATCC 12472) was employed to confirm the QSI activity of eugenol. Eugenol was purchased from Alfa-Aesar, USA, and stock solution was made by dissolving in 95% ethanol. These samples were then stored at −20 °C until further use. Working stocks were made from the stock solutions by diluting with required volume of deionized water. Soft agar overlay assay was performed to examine the QSI activity. Briefly, 5-mm diameter sterile discs were loaded with eugenol (0.3 mg) and placed over LB agar. A disk loaded with methanol was maintained as a control. The plates were then overlaid with 10 mL soft agar consisting of 0.1 mL of appropriately diluted (0.4 OD at 600 nm), freshly grown culture of *C. violaceum* ATCC 12472. Plates were incubated for 18 h at 30 °C to check for the presence of a violacein inhibition zone [18]. 

### 2.14. Data Analysis

Data were analyzed by using basic statistical methods with the tools in Microsoft Excel. Differences in data were evaluated by *t*-test analysis, and errors among replicates were expressed in the form of standard deviations. Unless otherwise stated, each experiment in this study was done in at least triplicate.

## 3. Results

### 3.1. Qualitative Screening for QSI Activity—Violacein Inhibition Assay

**Figure 1:**
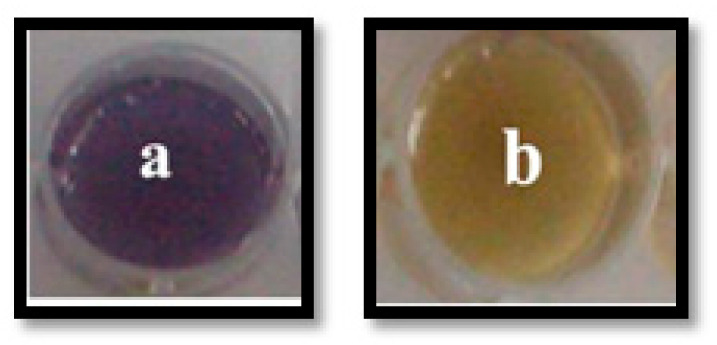
Inhibition of violacein production by *O. sanctum* extract. The images show violacein production by *C. violaceum* 12472 cultivated in the absence (**a**) and presence (**b**) of *O. santum* (12 mg/mL).

In our qualitative screening, the extracts of *O. sanctum* showed a remarkable ability to inhibit violacein production in *C. violaceum* (ATCC 12472), in a dose dependent manner and without affecting the bacterial growth. However, complete inhibition of violacein pigment production was observed at 12 mg/mL (Figure 1). Hence, the extracts of *O. sanctum* could be considered as an excellent source of QSI compounds.

### 3.2. Determination of MIC

It is well known that an efficient QSI agent should not have any growth inhibitory effect on bacteria. Therefore, the MIC for *O. sanctum* against test bacterial pathogens was determined. The minimum dosage of extract that inhibited the visible bacterial growth was recorded as the MIC. The MIC of *O. sanctum* extract against *E. coli*, *Vibrio harveyi*, *V. parahaemolyticus*, and *V. vulnificus* was recorded as 25 mg/mL, whereas against PAO1, *P. mirabilis*, and *S. marcescens*, an MIC value of 12.5 mg/ml was observed. These results are tabulated in Table 1. Notably, the concentrations below the MIC were considered as sub-MICs which were not anticipated to show any bactericidal activity. Hence, to analyze the QS-mediated antibiofilm potential of *O. sanctum*, sub-MICs such as 3, 6, 9, and 12 mg/mL for *O. sanctum* were chosen for QS related assays, including the inhibition of biofilm biomass and EPS production. 

### 3.3. Biofilm Biomass Quantification Assay

**Figure 2:**
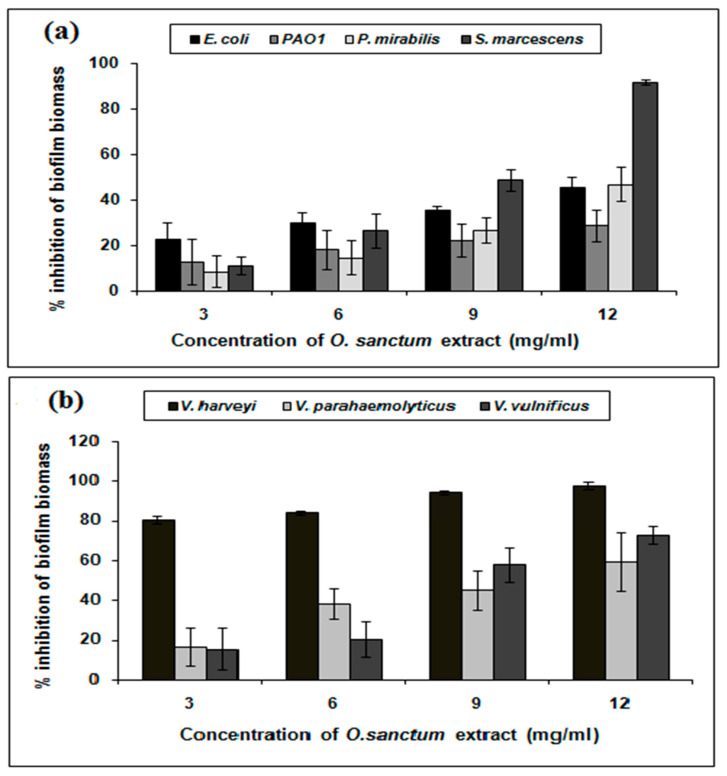
Inhibition of biofilm biomass by *O. sanctum* extract in Gram-negative human bacterial pathogens (**a**) and aquatic bacterial pathogens (**b**).

As QS regulates the biofilm formation of bacterial pathogens [22], the QS inhibitory potential of the *O. sanctum* extract was further investigated for its effects on the biofilm biomass production of test bacterial strains. The attained results displayed a dose dependent reduction in biofilm biomass production in *E. coli*, PAO1, *P. mirabilis*, and *S. marcescens* in the presence of *O. sanctum* extract (3–12 mg/mL), compared to the respective controls. At 12 mg/mL concentration, the *O. sanctum* extract showed a maximum 46%, 29%, 47%, and 92% reduction in *E. coli*, PAO1, *P. mirabilis*, and *S. marcescens*, respectively, as depicted in Figure 2a. Additionally, the extracts of *O. sanctum* showed pronounced antibiofilm activity against aquatic pathogens; the values were found to be 98%, 59%, and 73% for *V. harveyi*, *V. parahaemolyticus*, and *V. vulnificus*, respectively, at 12 mg/mL (Figure 2b). Nevertheless, the treatment of *O. sanctum* extracts did not reveal any inhibitory effect on planktonic growth.

### 3.4. Inhibition of Biofilm EPS

**Figure 3:**
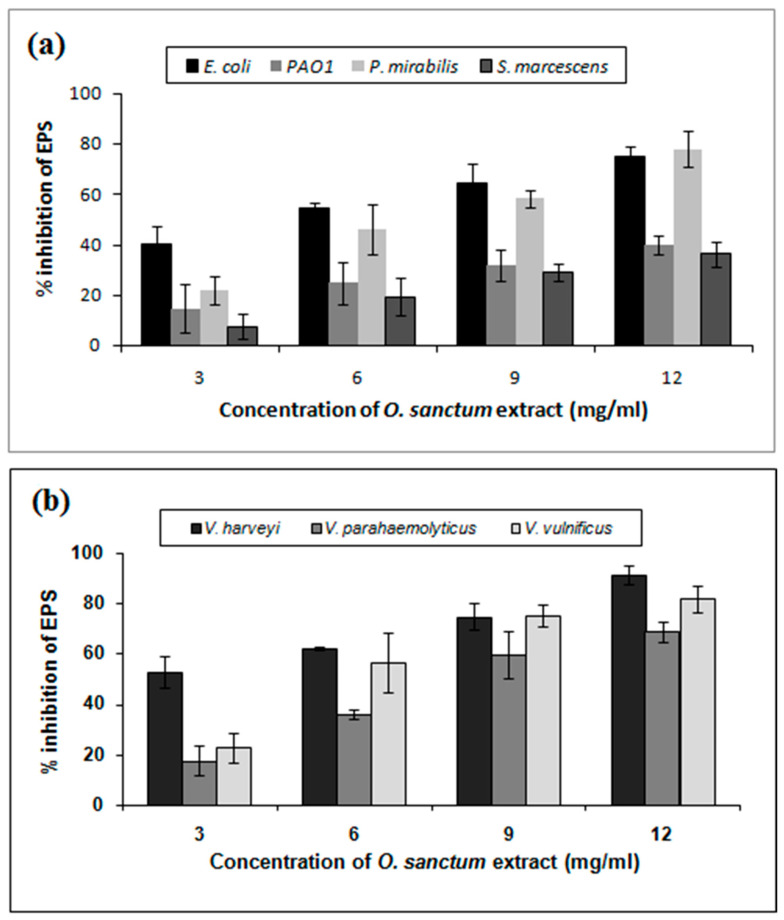
Inhibition of EPS production by *O. sanctum* extract in Gram-negative human bacterial pathogens (**a**) and aquatic bacterial pathogens (**b**).

Since EPS plays a crucial role in biofilm formation, the ability of *O. sanctum* extract to inhibit QS-mediated EPS production on target pathogens was examined. The biofilm EPS was extracted from *O. sanctum* treated and untreated control samples. As evidenced in Figure 3, treatment with increasing concentrations (3–12 mg/mL) of *O. sanctum* extracts decreased the EPS production compared to the control. At 12 mg/mL, maximum levels of inhibition were recorded as 75%, 40%, 78%, and 36% in human pathogens *E. coli*, PAO1, *P. mirabilis*, and *S. marcescens*, respectively, (Figure 3a), whereas 91%, 68%, and 82% reductions were recorded in aquatic pathogens *V. harveyi*, *V. parahaemolyticus*, and *V. vulnificus*, respectively (Figure 3b).

### 3.5. Antibacterial Assay

**Figure 4:**
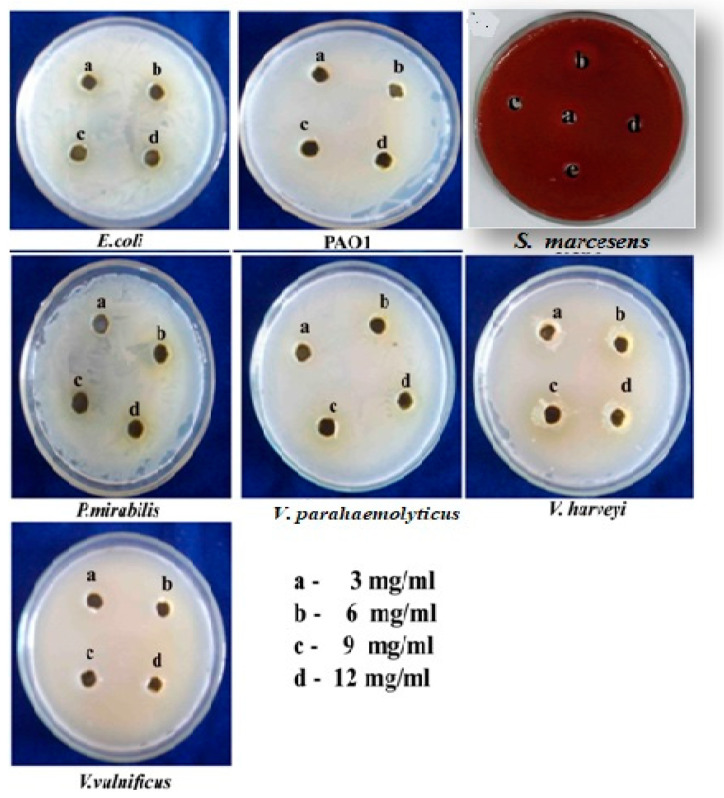
Effect of *O. sanctum* at test concentrations on the growth of bacterial pathogens by WDAA.

As shown in Figure 4, none of the bacteria displayed a zone of clearance around the wells containing *O. sanctum* extract at the test concentrations. Hence, it is suggested that the inhibition of biofilms and EPS by *O. sanctum* was achieved through the inhibition of QS rather than bacterial growth. 

### 3.6. GC-MS Analysis

**Figure 5:**
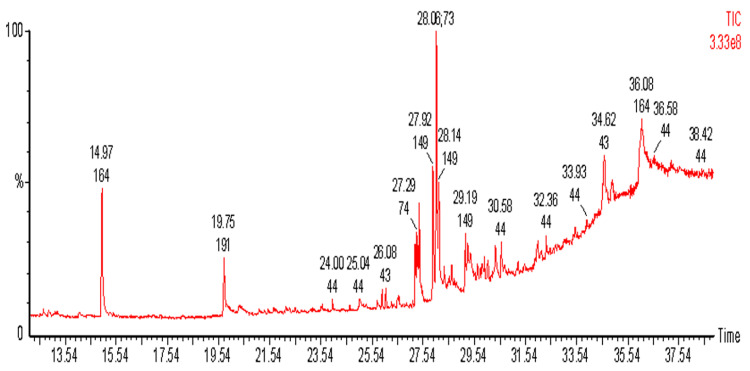
Biochemical analysis of *O. sanctum* extract through GC-MS analysis.

GC-MS analysis was done to identify the bioactive compounds present in the *O. sanctum* extract. The results unveiled the presence of sixteen different bioactive compounds in the *O. sanctum* extract (Figure 5), as tabulated in Table 2. The results of our GC-MS analysis evidenced the presence of acetyl eugenol and eugenol as the major constituents in the extract of *O. sanctum.*

### 3.7. Computational Studies

**Figure 6:**
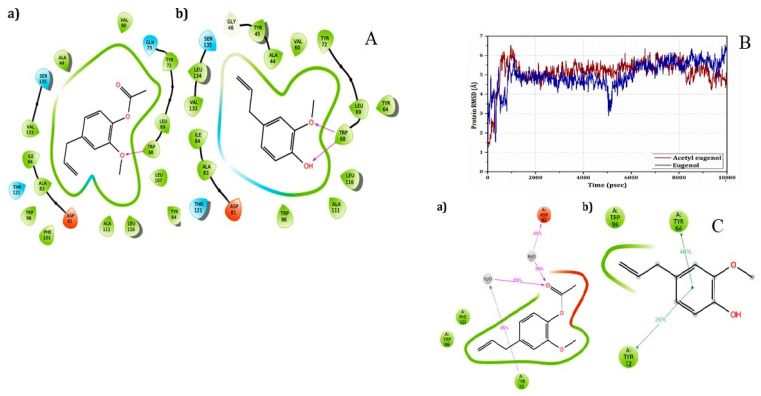
(**A**) 2D interaction plot profile of (**a**) acetyl eugenol and (**b**) eugenol with RhlR. (**B**) Root Mean Square deviation for the protein–ligand complexes calculated during the simulations. (**C**) Post-MD simulation interaction profile of the protein–ligand complexes of: (**a**) acetyl eugenol and (**b**) eugenol.

The x-ray crystal structure of the native complex of PqsE and RhlR with the synthetic antagonist mBTL of *E.coli* was found to be a suitable template to model the query sequence (P54292). The Modeller9v7 generated five models for the query sequence, and the model with the best DOPE score, i.e., −27,851.96, was selected. The quality of the generated model was found to be reliable, based on the Ramachandran plot statistics. Further, the docking carried out using Glide XP showed that both acetyl eugenol and eugenol had similar scoring functions and binding modes (Table 3). In the case of RhlR-acetyl eugenol, the oxygen atom of the compound showed H-bond interactions with the amine group of Trp68 (O…NH; bond distance = 1.70Å) (Figure 6A(a)), whereas in RhlR-eugenol, two H-bond interactions were observed, in which the oxygen and hydroxyl groups of the compounds showed H-bond interactions with Trp68 (O…NH, bond distance = 2.23 Å; OH…NH, bond distance = 2.14 Å) (Figure 6A(b)). Further, the stability of the protein–ligand complexes was studied via molecular dynamics simulation. It was observed that both the complexes showed initial deviations in the RMSD for the first few nanoseconds, and after that, stability in the remaining 100 nanoseconds (Figure 6B). During the simulation of RhlR-acetyl eugenol, two water-mediated H-bonds via Asp81 and Tyr72 were preserved at over 86% (Figure 6C(a)), whereas in the case of RhlR-eugenol, two pi–pi interactions with Tyr64 and Tyr72 were preserved at over 46% and 36%, respectively (Figure 6C(b)). The observed bonding interaction during the simulation plays a major role in the stability of ligands with RhlR. The results indicate the competitive inhibitory activity of eugenol and acetyl eugenol against RhlR receptors. Notably, eugenol displayed slighlty better activity than acetyl eugenol, and hence, further studies were performed with eugenol.

### 3.8. FT-IR Analysis

**Figure 7:**
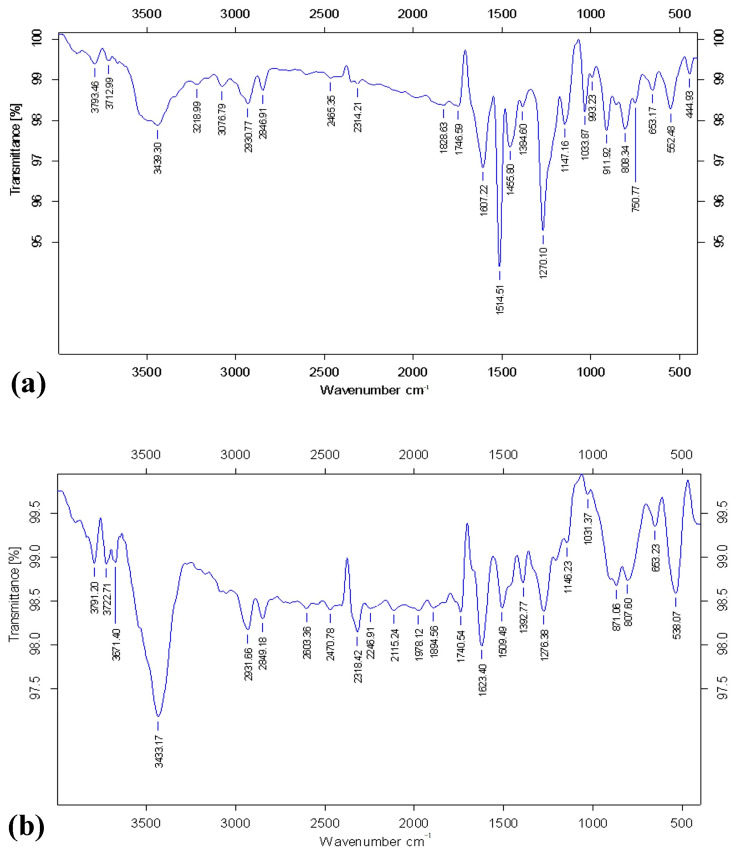
Comparison of the FTIR spectrua of active fraction F34 and eugenol. (**a**) FTIR profile of fraction F34. (**b**) FTIR profile of eugenol.

Since the major portion of the *O. sanctum* extract comprised eugenol, as evident from our GC-MS analysis (Figure 5, Table 2) and based on computational studies results (Figure 6, Table 3), the functional groups of commercially available eugenol and the active fraction obtained from the *O. sanctum* extract were compared through FTIR analysis. Interestingly, the functional group profile of the active fraction was comparable with that of commercial eugenol with 98% purity, and thus, it is suggested that the presence of eugenol and its derivatives was responsible for the QSI activity on the *O. sanctum* extract (Figure 7).

### 3.9. UV Spectra Analysis

**Figure 8:**
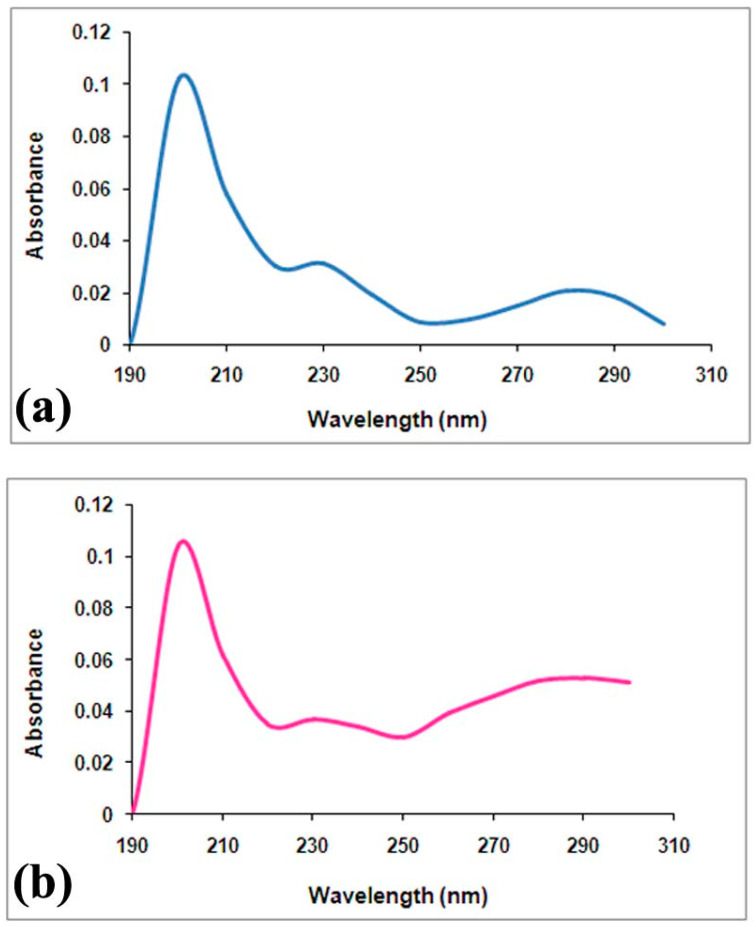
Determination of QSI compounds through UV spectrum analysis: (**a**) UV spectrum of fraction F34. (**b**) UV spectrum of the commercial eugenol.

The active fraction (F34) and purified eugenol (commercially available with 98% purity) were further compared through UV spectrum analysis. Interestingly, the UV spectra of the fraction 34 had most of the same peaks as the UV spectra of eugenol, i.e., two main absorption bands between 190 and 220 nm and 270 and 300 nm (Figure 8). These data suggest that the active fraction F34 of the *O. sanctum* extract contained eugenol as the major component, which may account for its QSI activity on *O. sanctum.*

### 3.10. Confirmation of the QSI Activity of Eugenol

**Figure 9:**
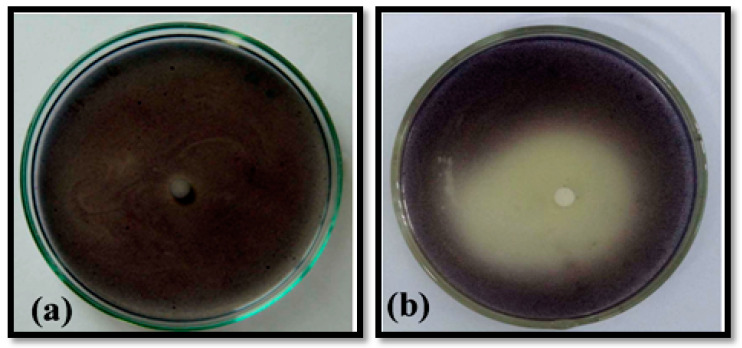
Confirmation of QSI activity in the identified compound eugenol using the biomarker *C. violaceum* 12472. (**a**) Untreated culture (**b**) Eugenol treated culture.

The obtained results clearly evidence the inhibition of pigment production around the discs loaded with eugenol (0.3 mg), without affecting the bacterial growth (Figure 9); hence, the QSI activity of eugenol was confirmed.

## 4. Discussion 

Globally, 70–80% of the world’s population depends on herbal medicine for their health concerns. Plants are being extensively assessed as prospective leads in drug development [23], as they have been recognized to contain a great range of attractive phytochemicals. These phytochemicals are generally recognized as safe (GRAS) and, in most cases, their use does not lead to any negative consequences [24]. It is also envisaged that phytochemicals with prominent antibiotic properties perhaps also have antipathogenic properties [12]. Phytochemicals with antipathogenic properties neither kill nor arrest bacterial growth; rather, they attenuate the bacterial virulence, and hence, do not allow the bacteria to develop resistance [25]. In this regard, the Indian traditional medicinal plant *O. sanctum* was examined for its antipathogenic activity against biofilm-forming pathogens.

It has been reported that medicinal plants contain several phytochemicals with QSI potential [12]. The present study evaluated the potential of *O. sanctum* as a source of antipathogenic compounds. Considering that biofilm formation is regulated by signal mediated QS mechanisms and that it confers resistance to bacteria [26], it is assumed that agents that block the QS mechanism rather than inhibiting bacterial growth may also effectively inhibit biofilm formation and could therefore prevent the emergence of bacterial resistance. As expected, in the present investigation, the extract at test concentrations effectively reduced the biofilm biomass without causing growth retardation. Correspondingly, a dose dependent inhibition of biofilm-associated behaviors has been observed with south Indian medicinal plants such as *C. cyminum* [16] and *C*. *spinosa* [15] at sub-MIC levels. Apart from biofilm formation, the extract examined in this study also affected the EPS secretion of all test pathogens; this was assumed to be linked with the development of bacterial biofilms [3].

The presence of biofilm EPS prevents the entry of antibiotics inside the biofilm matrix and renders the bacteria resistant to antibiotics [27]. So, it is envisaged that compounds that inhibit EPS may ultimately lead to the poor development of biofilms, making antibiotic treatment more effective by allowing the entry of antibiotics into the biofilm. Despite the fact that EPS has been synthesized via QS mechanisms in various bacteria, so far, the QSI activity of *O. sanctum* in terms of reducing EPS secretion has not been studied on the target pathogens used in this study; however, the use of some other medicinal plants has been proposed [15,16,28]. In the present study, upon treatment with *O. sanctum* extract, the production of EPS was reduced significantly relative to the control in all test pathogens. These observed reductions in EPS production in all test pathogens were positively correlated with biofilm biomass inhibition. Hence, the reduced levels of EPS might be attributed to poor biofilm development and a lower biomass. 

Although *O. sanctum* has been reported to have antibacterial properties, the extracts at tested concentrations (sub-MICs) were not found to be toxic against any of the bacteria. This was proved by the WDAA, in which no growth inhibitory zones were found around the wells. Together, the results indicated that the inhibitory effect of this extract on biofilm and EPS was due to interference with bacterial QS, rather than killing or inhibiting bacterial growth. Thus, it is envisaged that *O. sanctum* could be a potential source of QS inhibitors to interrupt cell signaling via different mechanisms, including reducing the activity of the signal cognate receptor protein, inhibiting the production of QS signal molecules, and targeting QS signals, either by degradation or mimicking as signal analogs [29]. 

The active principle responsible for the QSI activity of *O. sanctum* was characterized via conventional and chromatography techniques including GC-MS, column chromatography, and FTIR and UV spectrum analyses. As evidenced in Figure 5, GC-MS analysis of the *O. sanctum* extract revealed the presence of eugenol as the major component, although other bioactive compounds were present. Our investigation used column chromatography followed by FTIR and UV spectrum analyses. Among the eluted 46 fractions, fraction F34 was considered to be the active fraction regarding QSI activity against *C. violaceum*. In view of the presence of eugenol as a major component of *O. sanctum* extract, as evidenced by GC-MS analysis (Table 2) and computational studies (Figure 6, Table 3), the FTIR profile of active fraction F34 was compared with that of purified eugenol; the profiles were found to be highly comparable (Figure 7). These results were also supported by the results of our UV spectral analysis, in which the UV spectrum of active fraction F34 showed considerable similarity with the peak profile of purified eugenol (Figure 8). Together, the data evidenced the presence of eugenol in the active fraction F34 of our *O. sanctum* extract. Therefore, it is suggested that the QSI activity of *O. sanctum* might be due to the presence of the phenolic compound eugenol. The QSI activity of commercial eugenol in terms of reducing the QS-dependent violacein production in biomarker strains (Figure 9) was the basis of our conclusion in this regard. Despite the fact that eugenol in plant essential oils has known bactericidal activity against many pathogenic bacteria [30], reports on its QSI activity in terms of attenuating biofilm-associated behaviors are scarce. Regarding to the mode of action, the structure–activity relationship was studied to assess whether eugenol could interfere with the perception of signal molecules by their cognate receptors (Figure 6 and Table 3). Interestingly, eugenol showed structural similarities with AHL molecules, and it was envisaged that eugenol possibly competes with the AHL molecules in binding with receptors, which would result in the reduced expression of the QS system. To confirm the anti-QS activity of eugenol, commercially available eugenol was tested against biomarker strain *C. violaceum* 12472. Surprisingly, a substantial inhibition in violacein pigment was observed. Regarding the feasibility of extracting eugenol in large quantities from *O. sanctum*, it is worth noting that numerous extraction methods have been developed, including supercritical carbon dioxide extraction, steam distillation, and solvent extraction. Notably, the yield percentage of eugenol obtained from *O. sanctum* has been found to be good, with nearly no wastage. Commercially available eugenol is commonly obtained by steam distillation and solvent extraction [31]. Thus, *O. sanctum* serves as a significant natural source of eugenol, and various extraction methods have demonstrated the feasibility of obtaining eugenol in large quantities from this plant, presenting a viable alternative to the commercial production process. Additionally, both *O. sanctum* extracted eugenol and commercial eugenol were found to demonstrate equivalent antiviral activity against dengue virus [32]. The flavonoid compound catechin, derived from *C. albiflorum* bark extract, was reported to inhibit the PAO1 QS mechanism [33]. Recently, a Piper beetle derived bioactive compound, phytol, was found to mitigate QS regulated virulence factors and biofilm formation in *Serratia marcescens* [34]. Further investigation of the biofilm inhibition potential of *O. sanctum* extracts is obviously recommended to determine the QSI mechanism of such an extract.

## 5. Conclusions

Given the importance of developing new strategies to control biofilm development because of its association with several negative consequences in medical and aquaculture food producing settings, a traditional medicinal plant was investigated in terms of its biofilm inhibitory activity, as the pathogenesis of such bacteria is linked with the signal mediated QS mechanism, which facilitates the secretion of EPS by biofilm formation. The attained results revealed the ability of *O. sanctum* to mitigate QS-mediated EPS and biofilm formation in several human and aquatic pathogens without retarding bacterial growth. Hence, it is envisaged that the identification of the active principles in this extract will lead to the development of novel alternatives to antibiotics in order to address the emergence of resistance among pathogenic bacteria.

## Figures and Tables

**Table 1:** MIC of *O. sanctum* extract against target bacterial pathogens.

Target Pathogen	*O. sanctum* (mg/mL)
*E. coli*	25.0
PAO1	12.5
*P. mirabilis*	12.5
*S. marcescens*	12.5
*V. harveyi*	25.0
*V. parahaemolyticus*	25.0
*V. vulnificus*	25.0

**Table 2:** Components identified in the *Ocimum sanctum* leaf extract.

S. No.	Peak Name	Retention Time	%Peak Area
1.	1-(4-Hydroxymethylphenyl) ethanone	14.09	1.2087
2.	Eugenol	14.97	21.0757
3.	Phenol, 2,4-bis(1,1-dimethylethyl)-	19.75	6.7635
4.	D-Allose	20.36	0.6397
5.	1,2-Benzenediol, 4-(1,1-dimethylethyl)-	22.17	0.2880
6.	Z-2-Dodecenol	24.00	1.6557
7.	3,7,11,15-Tetramethyl-2-hexadecen-1-ol	25.92	1.3831
8.	2-Pentadecanone, 6,10,14-trimethyl-	26.08	1.6786
9.	1,2-Benzenedicarboxylic acid, butyl octyl ester	27.23	1.4609
10.	Benzenepropanoic acid, 3,5-bis(1,1-dimethylethyl)-4-hydroxy-, methyl ester	27.39	2.8220
11.	1,2-Benzenedicarboxylic acid, butyl 2-methylpropyl ester	27.92	11.8857
12.	n-Hexadecanoic acid	28.06	13.7495
13.	Octadecanoic acid	30.58	4.3143
14.	Benzene, 6-heptynyl-	32.02	2.2544
15.	16-Heptadecenal	32.36	2.3497
16.	Acetyl eugenol	36.08	26.4706

**Table 3:** Docking score and interaction profile of acetyl eugenol and eugenol with RhlR protein.

Protein	Compound	Glide Gscore Kcal/mol	Glide Energy Kcal/mol	Glide Emodel Kcal/mol	H Bond Interaction
RhlR	Acetyl eugenol	−6.192	−27.812	−34.097	Trp68(NH...O;1.70Å)
	Eugenol	−5.805	−28.081	−37.237	Trp68(NH...O;2.23Å); (NH...O;2.14Å)

## Data Availability

The data used to support the findings of this study are available from the corresponding author upon request.
